# Cucurbitacin-B instigates intrinsic apoptosis and modulates Notch signaling in androgen-dependent prostate cancer LNCaP cells

**DOI:** 10.3389/fphar.2023.1206981

**Published:** 2023-06-28

**Authors:** Ahmed Alafnan, Nasrin E. Khalifa, Talib Hussain, Mhdia Elhadi Osman

**Affiliations:** ^1^ Department of Pharmacology and Toxicology, College of Pharmacy, University of Ha’il, Ha’il, Saudi Arabia; ^2^ Department of Pharmaceutics, College of Pharmacy, University of Ha’il, Ha’il, Saudi Arabia; ^3^ Department of Pharmaceutics, Faculty of Pharmacy, University of Khartoum, Khartoum, Sudan; ^4^ Department of Clinical Pharmacy, College of Pharmacy, University of Ha’il, Ha’il, Saudi Arabia

**Keywords:** anti-cancer, apoptosis, cell cycle, cucurbitacin-B, Notch signaling

## Abstract

**Introduction:** Among numerous triterpenoids of the Cucurbitaceae family, Cucurbitacin-B (Cur-B) is being explored for its pharmacological attributes. Reports from previous studies have explicitly shown that Cur-B possesses substantial anticancer effects. The present report focuses on exploring the anticancer attributes of Cur-B against androgen-dependent PCa LNCaP cells.

**Methods:** LNCaP cells were exposed to commercially available purified Cur-B at varying concentrations that were selected as 5, 10, 15, 20, and 25 µM for some time of 24 h to perform various experimental studies.

**Results:** Cytotoxicity evaluation revealed that Cur-B impeded the LNCaP cell’s viability at 5 µM (*p* <0.05) which increased considerably at a concentration of 25 µM (*p* <0.001). Cur-B was also efficacious in inducing the changes within nu-clear morphology followed by a concomitant increase in the activities of key caspases including caspase-3, -8, and -9 intriguingly in a dose-dependent trend. Cur-B treatment not only resulted in the augmentation of intracellular ROS levels within LNCaP cells at 5 µM (*p* <0.05) but also in-creased significantly at 25 µM concentration (*p* <0.001). Elevation in the ROS levels was also found to be correlated with dissipated mitochondrial membrane potential (ΔΨm) which culminated in the onset of significant apoptosis at 25 µM concentration (*p* <0.001). Cur-B exposure also resulted in the downregulation of cyclin D1, cyclin-dependent kinase 4 (CDK4) followed by amplified levels of p21^Cip1^ mRNA. Importantly, exposure of Cur-B competently reduced the expression of the Notch signaling cascade which may be the plausible cause behind Cur-B-instigated apoptotic cell death and cell cycle arrest in LNCaP cells.

**Discussion:** These observations thus, explicitly indicated that Cur-B could be plausibly further explored as potent therapeutics against androgen-dependent PCa.

## 1 Introduction

Prostate cancer (PCa) is a debilitating malignancy within the male population globally. As per the recently available statistics, 1,414,259 male individuals were diagnosed positive for PCa by the end of the year 2020. Indeed, PCa contributed to 7.3% of the total 19,292,789 cases of positively diagnosed different cancers. Moreover, PCa was also responsible for 375,304 confirmed deaths globally by the end of the year 2020 (Bilal, et al., 2022). Usually, the onset of the disease in males happens during 60–70 years of age, which significantly increases both the mortality and morbidity of PCa (Alafnan, et al., 2021). The Notch signaling pathway serves as an important development pathway that modulates the onset and progression followed by metastasis of PCa (Leong and Gao, 2008). Previously, Notch signaling was reviewed to modulate the tumorigenicity of PCa stem cells and is thus involved in regulating the proliferation of oncogenes along with the development of chemotherapeutic resistance (Mourkioti, et al., 2022). Indeed, hyperactivated Notch signaling has been the hallmark of PCa observed in both clinical and preclinical models (Shou, et al., 2001; Mourkioti, et al., 2022).

Furthermore, Notch signaling was also shown to play an important role in imparting resistance toward enzalutamide within PCa cells that were found to be ameliorated by using Notch signaling inhibitors (Farah, et al., 2019). Exhaustive research over the last several decades has provided the impetus for a better understanding of some very important pathophysiological and their associated mechanisms in PCa. This has further contributed to elucidating various interventions (including surgical and/or radiological interventions usually coupled with chemotherapeutics) followed by androgen ablation therapies. Although effective, the efficiencies of these interventions are primarily dependent on the stage of PCa, thereby compelling the need for further exploration of new interventions against PCa. The present typical protocol for clinical management of prostate cancer mostly relies on the use of drugs like docetaxel, which have already been reported to cause a side effect on the health of individuals by affecting the microtubules and destabilizing them (Mukhtar, et al., 2014). Docetaxel has also been associated with various research studies to prompt resistance due to the instantaneous efflux of the therapeutic drug caused by mutations in the microtubules (Hwang, 2012).

In addition to it, another key drawback due to the utilization of standard chemotherapeutics is their related ill effects, which appallingly have an unconstructive impact on blood cells along with cells of the mouth and digestive tract and even hair follicles (Altun and Sonkaya, 2018). Among diverse recognized fruit and vegetable crops, Cucurbitaceae is one of the major families, which has almost 125 genera besides 960 species. The members of this family have been identified as a component of prehistoric medical and culinary traditions. Interestingly, they have also been considered a key component of folk medicines that are formerly mentioned in Ayurveda for their medicinal benefits (Olarewaju, et al., 2021). Quite a few members of the Cucurbitaceae family have already been mentioned for the treatment of various diseases. The chemical composition of cucurbitacins is referred to as triterpenoid adjoined by a base nuclear frame as 19-(10″9)-abeo-5α-lanostane that is responsible for carrying oxygen atoms at different positions (Kaushik, et al., 2015). A recent study has documented the antitumor effect of cucurbitacin compound and its derivative against human breast cancer cells like MCF-7 and MDA-MB 231 (Varela, et al., 2022). Cucurbitacin B (Cur-B) is a predominantly distributed natural compound. It primarily belongs to the families Cucurbitaceae and Cruciferae and has been shown in a range of plants, such as *Cucumis melo*, *Cucurbita andreana*, *Ecballium elaterium*, *Wilbrandia ebracteata*, and *Trichosanthes cucumerina* (Cai, et al., 2015). An accrued number of reports have demonstrated the multiple pharmacological activities, such as anti-inflammatory, antioxidant, antiviral, hypoglycemic, hepatoprotective, neuroprotective, and anti-cancer effects, which mainly contribute to the treatment and management of various ailments, namely, inflammatory diseases, neurodegenerative diseases, diabetes mellitus, and cancers (Chen, et al., 2008). It is worth noting that Cur-B is the most abundant and active form of cucurbitacin, and as a result, it has garnered substantial attention from researchers across the globe as compared to the other classes of cucurbitacin (Sallam, et al., 2018).

Nevertheless, the mechanistic approach to the anti-cancerous potential of Cur-B and its probable role in the modulation of the Notch signaling cascade remains unfamiliar in the case of androgen-dependent human PCa LNCaP cells. Therefore, we hypothesized this study to examine that Cur-B mediates anti-cancer effects through the modulation of Notch signaling components against human PCa LNCaP cells.

## 2 Materials and methods

### 2.1 Materials

Cucurbitacin-B (SKU: PHL82226) and inhibitors of respective caspases, propidium iodide (PI), DCFH-DA, and Hoechst-33342 dye were commercially procured from Sigma-Aldrich (St. Louis, Missouri, USA). The caspase-specific kits used in the study were procured from BioVision, Waltham, Massachusetts, United States. Materials required for tissue culture including Ham’s F12K media, FBS along with antibiotic–antimycotic solution, SYBR Green kit (DyNAmo ColorFlash), and Verso cDNA Synthesis Kit were obtained from Thermo Fisher Scientific, Waltham, Massachusetts, USA. MTT dye along with the total RNA purification kit was obtained from HiMedia Labs, Pune, India.

### 2.2 Cell culture

Human-derived androgen-dependent prostate adenocarcinoma LNCaP cells were obtained from the National Centre for Cell Science, Pune, India. LNCaP cells were allowed to proliferate under standard tissue culture conditions at 37 °C along with 5% CO_2_. The RPMI-1640 media supplemented with a cocktail of 1% antibiotic–antimycotic and 10% FBS was used during the study. The passage number of the LNCaP cells used in the present study was five. All the imaging reported in the present study was accomplished using FLoid Imaging Station (Thermo Fisher Scientific, United States).

### 2.3 Methods

#### 2.3.1 Cur-B-mediated cytotoxicity

The evaluation of cytotoxicity of Cur-B at varied concentrations on LNCaP cells (androgen-sensitive prostate adenocarcinoma) was performed through the MTT assay, with a slightly modified protocol (Marignol, et al., 2013). Approximately 5 × 10^3^ cells per well were given treatment of Cur-B at the concentration of 5, 10, 15, 20, and 25 μM for a time period of 24 h, incubated in a humidified environment. After incubation, the media of each well were removed, and MTT dye was added to it (5 mg/mL; 10 μL) and was further left for 4 h. The formazan crystals were dissolved in 100 μL DMSO, and the absorbance of treated and untreated groups was documented at 570 nm with the help of a spectrophotometer. The cellular viability of LNCaP cells was estimated as percentage (%) in comparison with the control LNCaP cells.

Cellular viability % = (absorbance of treated LNCaP cells)/(absorbance of untreated LNCaP control cells) × 100.

#### 2.3.2 Morphological attributes of PCa cells

Cur-B-treated PCa cells were observed for modifications in their morphology in comparison with untreated cells. Nearly 5 × 10^3^ cells/well were subjected to the treatment of Cur-B at previously mentioned concentrations and left for 24 h. Finally, morphological modifications in different groups of PCa LNCaP cells were examined in the bright light of FLoid Imaging Station.

#### 2.3.3 Effects on nuclear morphology

The apoptotic efficacy of Cur-B was evaluated on LNCaP cells, and the treated cells were examined qualitatively through DAPI (Tiwari, et al., 2021). LNCaP cells were seeded approximately 5 × 10^3^ cells/well. Cells were afterward exposed to the previously mentioned Cur-B concentrations, followed by an incubation of 24 h. Subsequently, the LNCaP cells were washed using chilled PBS. Thereafter, chilled methanol was used for fixing the LNCaP cells approximately for 10 min. Finally, these were then permeabilized using a mixture of 3% paraformaldehyde (PFA) and Triton X-100 (0.25%) and consequently stained with DAPI. The LNCaP cells were observed, and photomicrographs were captured at an excitation : emission ratio of 390/40 nm : 446/33 nm, respectively.

#### 2.3.4 Onset of apoptosis

For the assessment of Cur-B in instigating the apoptosis induction in LNCaP cells, PI staining was performed along with the quantification through ImageJ software (NIH, USA). Approximately 5 × 10^3^ cells per well were exposed to the treatment of Cur-B at previously mentioned concentrations and left for 24 h. The PI staining fluorescence was further recorded at an excitation: emission wavelength of 586/15 nm: 646/68 nm, respectively, and further quantified though ImageJ software.

#### 2.3.5 Estimation of caspase/s activities

Commercially accessible caspase-8, -9, and -3 colorimetric kits were acquired and utilized to exhibit the activities of stated caspases in androgen-sensitive LNCaP cells as per the supplier’s instructions. After Cur-B treatment (aforementioned concentrations), 3 × 10^6^ LNCaP cells were lysed using 0.05 mL chilled lysis buffer along with 10 min incubation on ice. The lysate was subjected to centrifugation at 4 °C, 10,000 rpm for 1 min, and the supernatant was collected. Thereafter, 0.05 mL lysate of treatment and control cells well supplemented with 0.05 mL reaction buffer. Subsequently, each well was further supplemented with DEVD-pNA (4 mM) and incubated for approximately 10 min; last, the absorbance of each well was recorded at 405 nm. Percentage enhancement within the activities of the different caspases was further pre-meditated by comparing the alterations with that of untreated LNCaP cells.

#### 2.3.6 Effects of caspase inhibitors

Cytotoxicity instigated by Cur-B on LNCaP cells was further investigated through using inhibitors for caspase-8, -9, and -3. LNCaP cells were at first treated for approximately 2 h with ZIETD-FMK, Z-LEHD-FMK, and Z-DEVD-FMK (50 μM each; inhibitors for caspase-8, -9, and -3, respectively). Afterward, the cells were retreated with Cur-B at aforementioned concentrations and incubated for another 24 h. Finally, the viable percentage of Cur-B-treated LNCaP cells was quantified following the procedure described previously in MTT-mediated estimation of viable LNCaP cells.

#### 2.3.7 Estimation of mitochondrial membrane potential (ΔΨm)

ΔΨm of Cur-B treated LNCaP cells was examined using commercially accessible kit using the supplier’s protocol. The fluorescence intensity was assessed approximately 30 min after addition to the Mito-NIR dye and assay buffer-B at excitation/emission 640/680 nm, respectively, through a microplate reader.

#### 2.3.8 ROS estimation

The elevation of ROS production after the Cur-B treatment within the androgen-sensitive LNCaP cells was assessed qualitatively with the help of DCFH-DA stain as described earlier (Ahmad, et al., 2021). Approximately 5 × 10^3^ cells per well were supplemented with Cur-B at aforementioned concentrations (5–25 μM) followed by 12 h of incubation. After incubation, the media from every well was removed, cells were stained using DCFH-DA (10 μM) and incubated in the dark for 30 min; last, the cells were washed carefully and visualized, and photomicrographs were captured under the green channel fluorescence microscope.

#### 2.3.9 Real-time qPCR analysis

For evaluating the alterations within the genes like CDK4, p21^Cipl^, cyclin D1, Bax, Bad, Bcl2, Notch1 , Hes1, and Jagged1, LNCaP cells (1 × 10^6^ cells/well) were transferred and left to adhere for the night under optimum tissue culture environment. After overnight adherence, these cells were given treatment with the previously stated Cur-B concentration for 24 h before their total RNA was extracted. The extracted RNA was further used to synthesize cDNA, and then the qRT-PCR-based analysis was performed using the SYBR Green qPCR Kit as per the supplier’s manual. Comparative CT method was employed for analyzing the variations in the expression of stated genes with GAPDH as the control gene. The primers used were the same as reported previously as demonstrated in Table 1 (Ahmad, et al., 2022; Alafnan, et al., 2022).

**TABLE 1:** List of forward and reverse sequences of primers used in the study.

Target gene	Forward (5′–3′) sequence	Reverse (3′–5′) sequence
CDK4	CCA​TCA​GCA​CAG​TTC​GTG​AGG​T	TCA​GTT​CGG​GAT​GTG​GCA​CAG​A
p21^Cip1^	AGG​TGG​ACC​TGG​AGA​CTC​TCA​G	TCC​TCT​TGG​AGA​AGA​TCA​GCC​G
Cyclin D1	CTT​CCT​CTC​CAA​AAT​GCC​AG	AGA​GAT​GGA​AGG​GGG​AAA​GA
Bax	TCA​GGA​TGC​GTC​CAC​CAA​GAA​G	TGT​GTC​CAC​GGC​GGC​AAT​CAT​C
Bad	CCA​ACC​TCT​GGG​CAG​CAC​AGC	TTT​GCC​GCA​TCT​GCG​TTG​CTG​T
Bcl2	ATC​GCC​CTG​TGG​ATG​ACT​GAG​T	GCC​AGG​AGA​AAT​CAA​ACA​GAG​GC
GAPDH	CGA​CCA​CTT​TGT​CAA​GCT​CA	CCC​CTC​TTC​AAG​GGG​TCT​AC
Hes1	GGA​AAT​GAC​AGT​GAA​GCA​CCT​CC	GAA​GCG​GGT​CAC​CTC​GTT​CAT​G
Notch1	CTGGTCAGGGAAATCGTG	TGG​GCA​GTG​GCA​GAT​GTA​G
Jagged1	TGC​TAC​AAC​CGT​GCC​AGT​GAC​T	TCA​GGT​GTG​TCG​TTG​GAA​GCC​A

#### 2.3.10 Statistical evaluation

Data characterize the mean ± SEM of separate experiments (n = 3), where each experiment was performed at least thrice. Statistical significances in between the formulated groups were estimated using one-way ANOVA.

## 3 Results

### 3.1 Cur-B suppressed the proliferation of androgen-dependent PCa LNCaP cells

**FIGURE 1:**
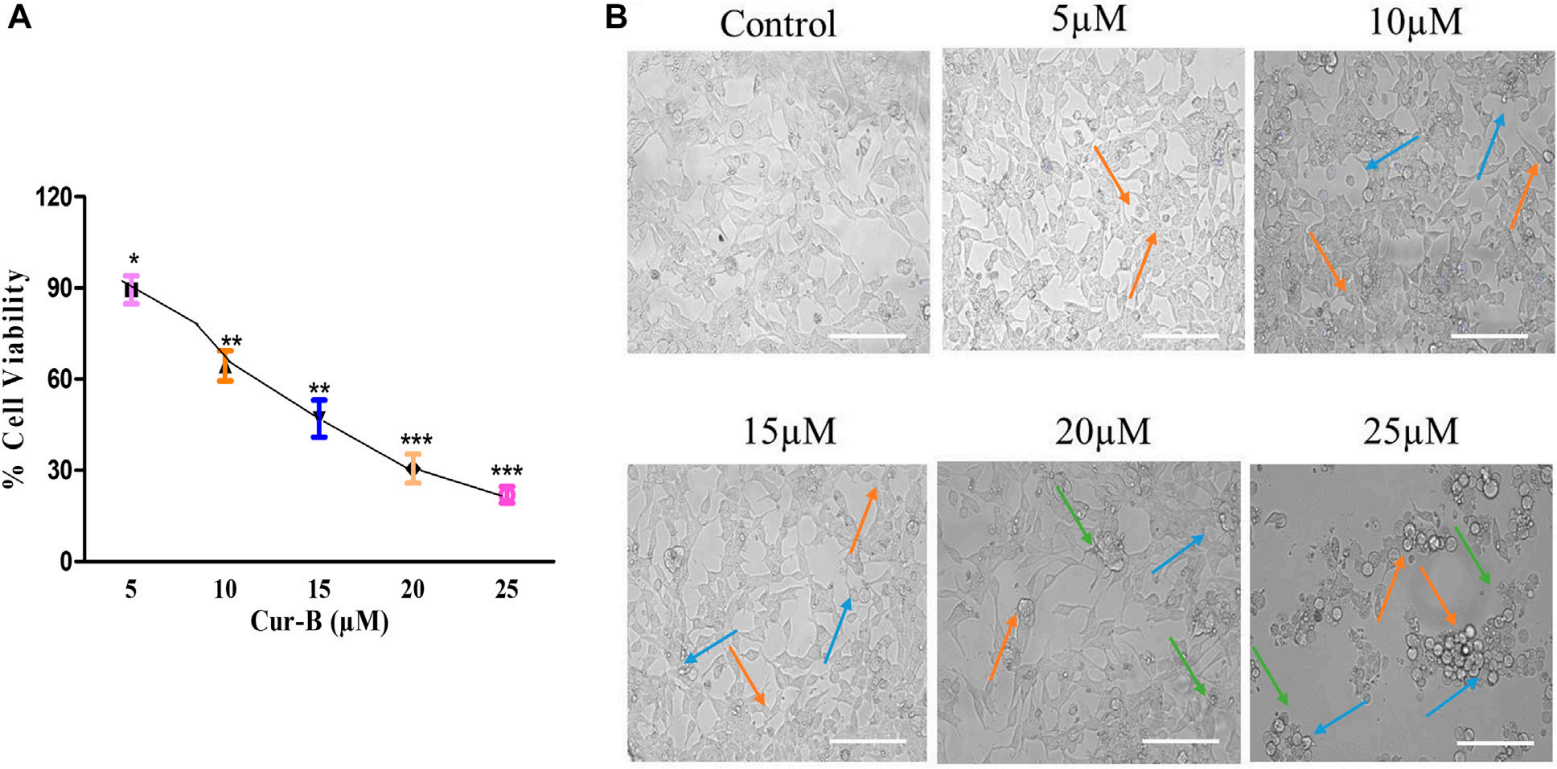
**(A)** Percent (%) cell viability of LnCap cells after treatment with various doses of Cur-B and **(B)** Alterations in morphology of LnCap cells upon treatment with different doses of Cur-B for 24 h as compared to the control. Scale bar = 100 µm.

The cytotoxicity assay was performed by treating LNCaP cells with different doses of Cur-B (5, 10, 15, 20, and 25 µM) for 24 h. IC50 of Cur-B against LNCaP cells was found to be 10.71 ± 1.08 µM (Figure 1A). It was demonstrated that the treatment of Cur-B substantially inhibited the proliferation of PCa cells (Figure 1B), which was found to 89.46% ± 3.75%, 64.42% ± 4.05%, 47.06% ± 4.97%, 30.59% ± 3.85%, and 21.92% ± 2.21% as compared to the vehicle or control at the suggested doses of 5, 10, 15, 20, and 25 µM of Cur-B, respectively. Thus, these findings concluded that Cur-B suppressed the proliferation rate of PCa cells in a dose-dependent manner.

### 3.2 Cur-B altered the morphology of androgen-dependent PCa LNCaP cells

To investigate whether the suppression of cell proliferation and occurrence of cell death are due to the treatment of Cur-B, LNCaP cells were examined under a phase contrast microscope. It was observed that treatment with increasing doses of Cur-B reduced the number of adherent cells and enhanced the number of floating PCa cells. Phase contrast micrographs revealed that a considerable number of cells underwent various morphological alterations, such as cell swelling, rounding up of cells, and disintegration of plasma, as shown in Figure 1C, substantiating Cur-B-mediated antiproliferative effects in androgen-dependent PCa cells, whereas vehicle or control cells exhibited unaltered flattened morphology.

### 3.3 Cur-B induced fragmentation and condensation in androgen-dependent LNCaP cells

**FIGURE 2:**
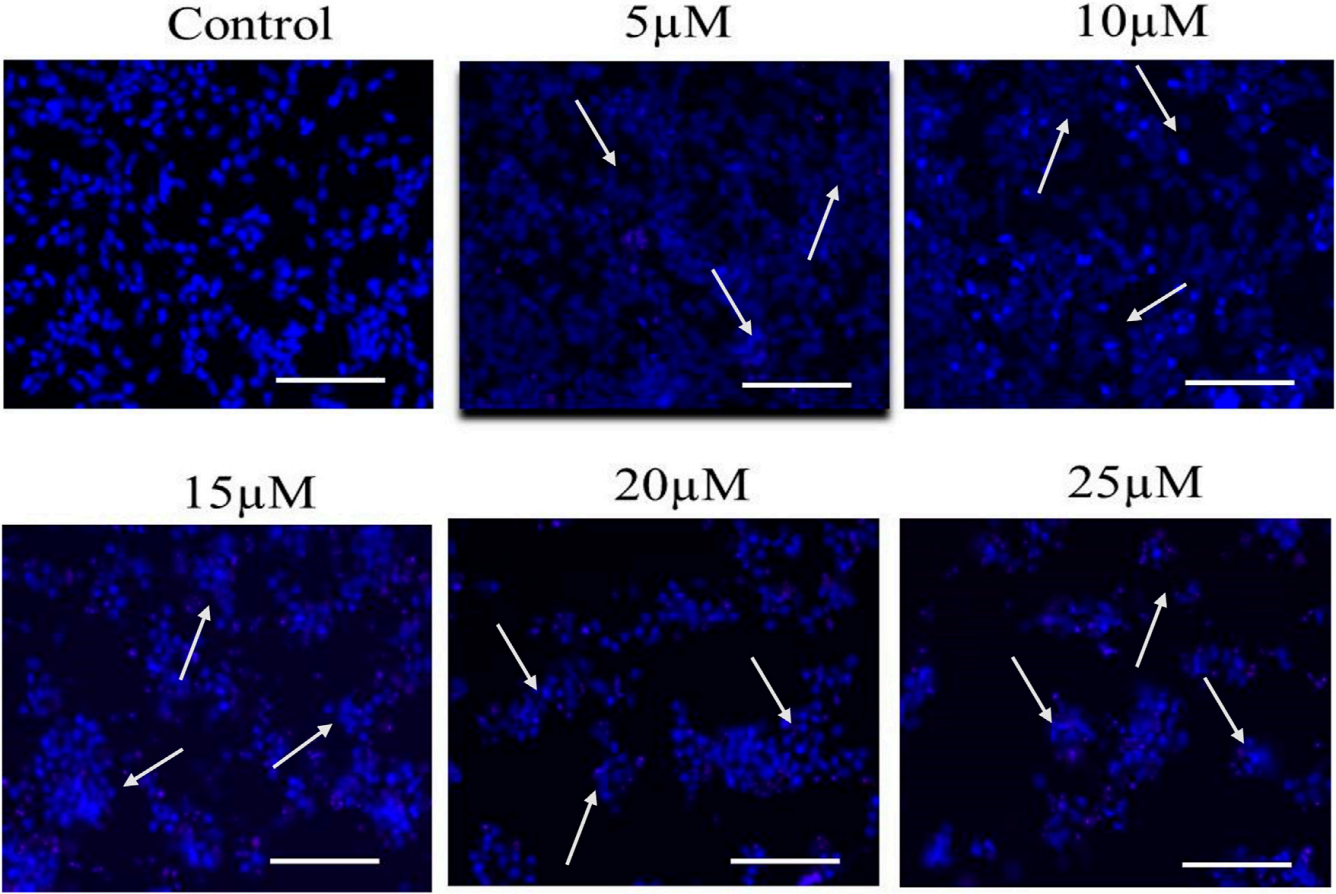
Cur-B mediates nuclear condensation and fragmentation in LNCaP PCa cells after staining with DAPI. White arrows indicate the nuclear condensation in LNCaP cells. Scale bar = 100 µm.

Multiple morphological alterations are found linked with the cells undergoing apoptosis such as the rounding up of cells that shrunk and lost communication with neighboring cells. However, there are some highly sensitive cells that even get removed from the surface of the plates. To evaluate nuclear condensation and fragmentation induced by Cur-B in LNCaP, cells were cultured with Cur-B for 24 h. The cells were stained with DAPI after treatment with Cur-B for 24 h and studied by fluorescence microscopy. It was evident that the treated PCa cells exhibited substantial condensation and fragmentation, which are peculiar attributes of apoptosis as compared to the vehicle or control, which demonstrated the exact round nuclei with diffuse blue fluorescence (Figure 2).

### 3.4 Cur-B instigated apoptosis in androgen-dependent PCa cells

**FIGURE 3:**
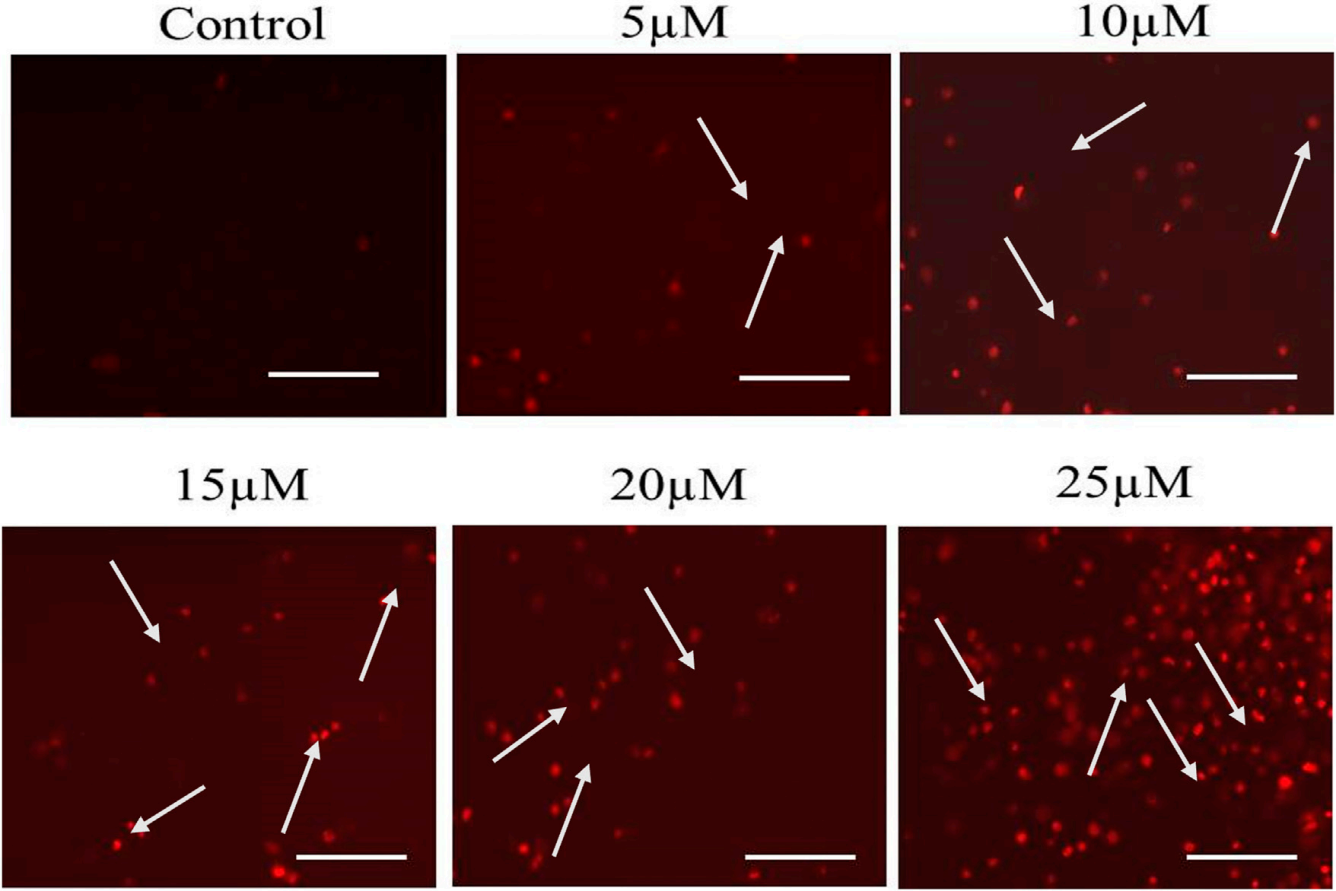
Cur-B mediated the instigation of apoptosis within LNCaP cells as evaluated through PI staining. White arrows indicate the apoptotic nuclei of Cur-B-treated LNCaP cells. Scale bar 100 µm. Significance among different dosage groups were determined using one-way ANOVA followed by Dunnett’s post-test, where * represents *p* < 0.05, **p < 0.01, and ***p < 0.001 (GraphPad Prism, Ver. 5).

Cur-B instigated apoptosis within LNCaP cells and was measured by PI staining through a fluorescence microscope. As shown in the fluorescence micrographs of Figure 3, apoptotic cells were distinguished by augmented red fluorescence suggesting the formation of apoptotic bodies in Cur-B-treated LNCaP cells. Indeed, due to the presence of intact nuclei in vehicle or control cells, they were defined by the presence of diffuse red fluorescence. Furthermore, Cur-B-induced apoptosis was quantified in LNCaP cells. As observed in Figure 3, a dose-dependent increase in apoptotic cells exhibiting bright red fluorescence was observed in comparison with the control. Thus, our results suggested that Cur-B-induced morphological alterations in a dose-dependent manner in the nucleus eventually lead to apoptosis.

### 3.5 Cur-B treatment increases caspase activation

**FIGURE 4:**
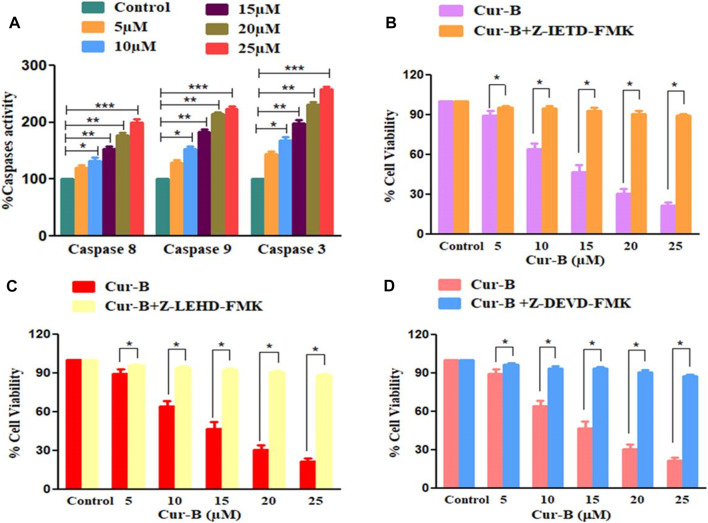
**(A)** Effect of Cur-B on the activation of caspase-8, -9, and -3 and **(B–D)** cytotoxic effects of Cur-B against the caspase inhibitor pre-treated LNCaP cells. Significance among different dosage groups were determined using one-way ANOVA followed by Dunnett’s post-test and by two-tailed Paired Student’s *t*-test as applicable, where * represents *p* < 0.05, **p < 0.01, and ***p < 0.001 (GraphPad Prism, Ver. 5).

To investigate, the underlying mechanism in Cur-B-induced apoptosis is due to the activation of caspases in PCa cells. As shown in Figure 4A, there was a substantial induction of caspase activities within PCa cells upon treatment with increasing doses of Cur-B (5, 10, 15, 20, and 25 µM) for 24 h. The percentage (%) increase in the activities of caspase-8 and -9 was found to be 19.87% ± 3.45%, 32.43% ± 4.19%, 52.72% ± 3.53%, 77.37% ± 3.57%, and 99.26% ± 4.96%; 29.65% ± 3.54%, 53.73% ± 3.40%, 83.58% ± 3.45%, 114.51% ± 2.36%, and 123.68% ± 3.64% in comparison with control, at the indicated doses of 5, 10, 15, 20, and 25 µM of Cur-B, respectively (Figure 4A). In addition, enhancement in caspase-3 activity was found to be 35.87% ± 3.45%, 54.43% ± 4.54%, 79.72% ± 4.72%, 123.96% ± 3.36%, and 173.68 ± 5.12, respectively. Thus, these findings concluded the involvement of both extrinsic and intrinsic pathways of apoptosis in Cur-B-treated PCa cells.

### 3.6 Attenuation of Cur-B-mediated apoptosis

To subsequently affirm the involvement of caspase activation in Cur-B-mediated apoptosis in LNCaP cells, the MTT assay was performed in prostate cells pre-treated with caspase-8, -9, and -3 inhibitors (Z-IETD-FMK, Z-LEHD-FMK, and Z-DEVD-FMK, respectively) for 2 h followed by treatment with different doses of Cur-B for another 24 h (Figures 4B–D). It was witnessed that preliminary treatment with caspase inhibitors completely attenuated the apoptosis induced by Cur-B in PCa cells suggesting that caspase activation is tightly linked to apoptosis induction in LNCaP cells.

### 3.7 Cur-B impeded ΔΨm

**FIGURE 5:**
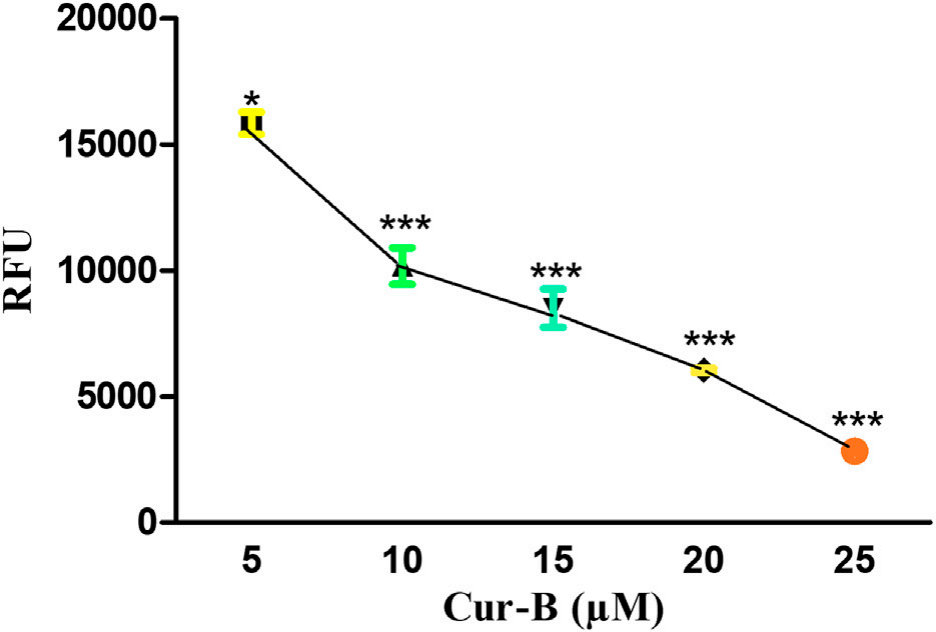
Cur-B mediates the decrease in NIR fluorescence in LnCap cells as compared to the control after staining with Mito-NIR dye indicating depolarized mitochondria. Scale bar 100 µm.

The loss in mitochondrial viability is an established mediator of apoptosis. The alteration within ΔΨm indicates the initiation of apoptotic cell death through the intrinsic pathway. As explicitly seen in Figure 5, Cur-B exposure succeeded in reducing Mito-NIR-mediated fluorescence, clearly outlining the efficacy of Cur-B in altering the ΔΨm in LNCaP cells comparatively with untreated control. Intriguingly, the ΔΨm reduction in LNCaP cells was directly proportional with the Cur-B concentration.

### 3.8 Cur-B increased intracellular ROS

**FIGURE 6:**
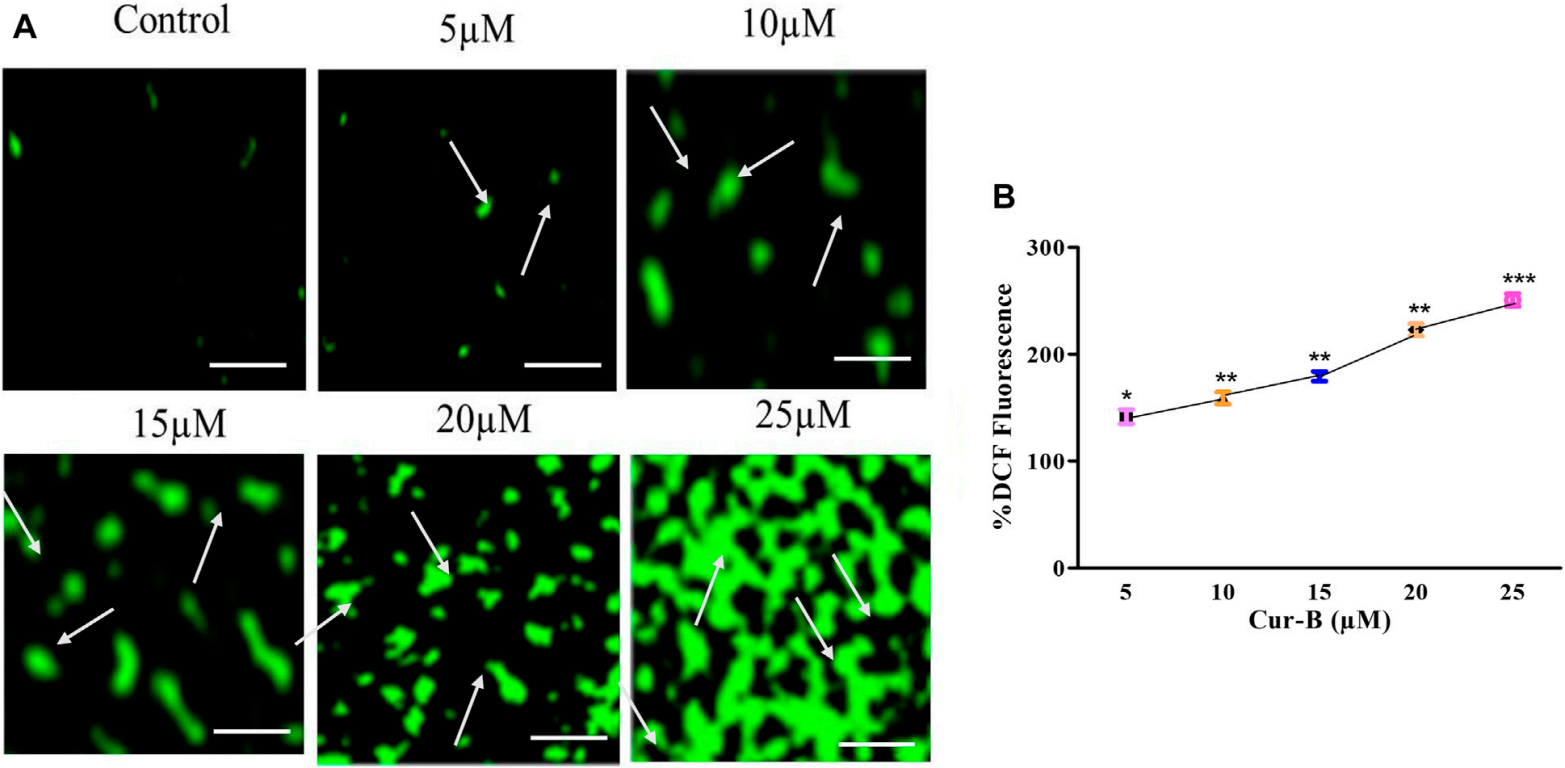
**(A)** Qualitative and **(B)** quantitative assessment of ROS levels within LnCap cells after treatment with various concentrations of Cur-B as compared to the control. Scale bar = 100 µµm.

To delineate the mode of action in Cur-B, we therefore assessed the levels of intracellular ROS in Cur-B treated and untreated PCa cells by the DCFH-DA staining method. It was observed that the aggregation of DCHF-DA in treated cells indicated that Cur-B elevated the ROS generation in LNCaP cells (Figure 6A). Furthermore, the quantitative assessment of ROS generation was performed to validate the aforementioned results. As observed, in LNCaP cells, the intracellular level of ROS was enhanced by 41.71% ± 5.48%, 59.01% ± 4.59%, 79.42% ± 3.62%, 122.88% ± 4.66%, and 150.86% ± 4.94% in C33A cells at the indicated doses of 5, 10, 15, 20, and 25 µM Cur-B, respectively (Figure 6B). Therefore, these results suggested that Cur-B elevated the production of ROS in PCa cells in a dose-proportional trend.

### 3.9 Cur-B modulated the expression of key gene mRNA in androgen-dependent LNCaP cells

**FIGURE 7:**
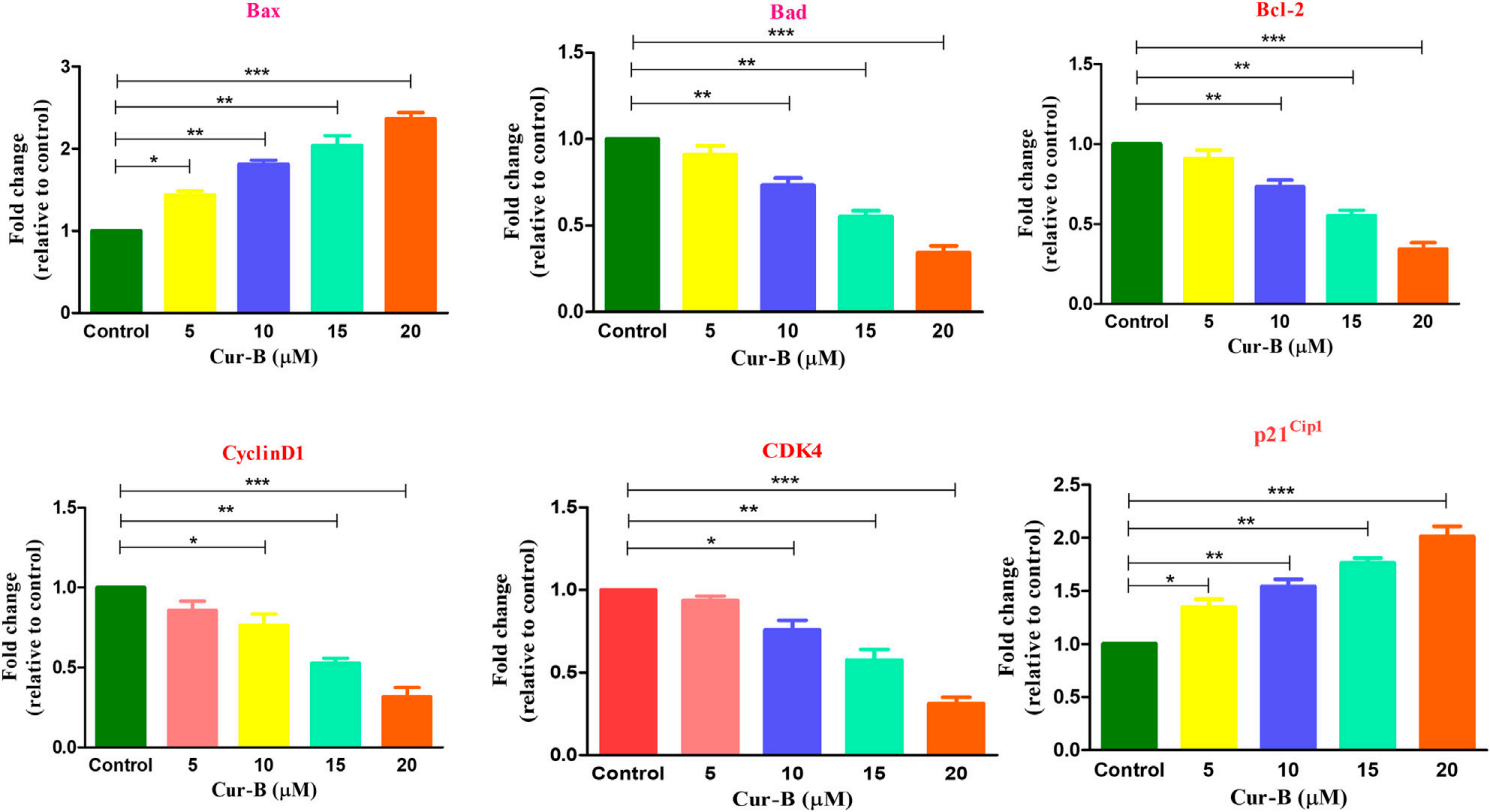
Effect of Cur-B in the modulation of apoptotic and cell cycle genes mRNA expression in LNCaP PCa cells. Significance among different dosage groups were determined using one-way ANOVA followed by Dunnett’s post-test where * represents *p* < 0.05, **p < 0.01, and ***p < 0.001 (GraphPad Prism, Ver. 5).

Real-time PCR analysis was performed to assess the effects of Cur-B on the gene expression of key genes of the cell cycle in LNCaP PCa cells. As shown in Figure 7, treatment with increasing doses of Cur-B decreased the mRNA expression of cyclinD1 and CDK4 to 0.85 ± 0.04, 0.76 ± 0.05, 0.52 ± 0.02, and 0.31 ± 0.04 folds; 0.93 ± 0.02, 0.76 ± 0.04, 0.57 ± 0.05, and 0.31 ± 0.03 folds, respectively, as compared with the control. In addition, the mRNA expression of p21^Cip1^ was augmented by 1.34 ± 0.06, 1.54 ± 0.05, 1.76 ± 0.03, and 2.09 ± 0.05 folds, as compared to the control. Thus, it is suggested that Cur-B significantly modulated the expression level of cell cycle-related genes in PCa cells.

### 3.10 Cur-B modulated the mRNA expression of pro- and anti-apoptotic proteins

To investigate the effect of Cur-B on the expression of apoptotic and anti-apoptotic markers in androgen-dependent LNCaP PCa cells, qPCR was performed. It was found that the treatment, with increasing concentrations of Cur-B (5, 10, 15, and 20 µM), declined in the gene expression of the anti-apoptotic marker, namely, Bcl2 by 0.91 ± 0.04, 0.73 ± 0.03, 0.55 ± 0.02, and 0.34 ± 0.03 folds. On the other hand, Cur-B treatment augmented the mRNA expression of Bax and Bad by 1.43 ± 0.03, 1.81 ± 0.04, 2.04 ± 0.09, and 2.36 ± 0.06 folds; and 1.32 ± 0.03, 1.53 ± 0.06, 1.79 ± 0.04, and 1.97 ± 0.05 folds, respectively, after 24-h treatment (Figure 7).

### 3.11 Cur-B mediates the downregulation of Notch signaling pathway in LNCaP cells

**FIGURE 8:**
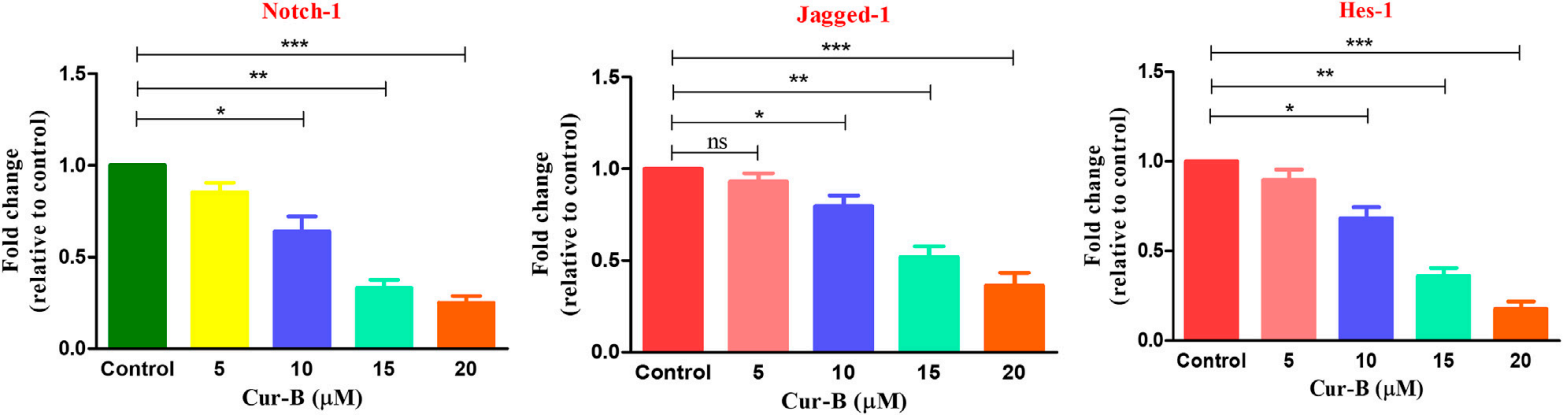
Effect of Cur-B on the key components of the Notch signaling pathway in LNCaP PCa cells. Significance among different dosage groups were determined using one-way ANOVA followed by Dunnett’s post-test, where * represents *p* < 0.05, **p < 0., and ***p < 0.001 (GraphPad Prism, Ver. 5).

To decipher the underlying molecular mechanism of Cur-B-mediated apoptosis in androgen-dependent LNCaP cells, its efficacy on modulating the key components of the Notch signaling cascade was studied. We examined the effect of Cur-B on Notch-1, its ligand Jagged-1, and its downstream target Hes-1 in LNCaP cells. As demonstrated in Figure 8, Cur-B reduced the mRNA expression of Notch-1 mRNA within LNCaP cells in a dose-proportional manner. The increasing doses of Cur-B declined the expression of Notch-1 by 0.85 ± 0.04, 0.64 ± 0.06, 0.33 ± 0.03, and 0.25 ± 0.03 folds, as compared to the vehicle or control (Figure 8). Additionally, we also studied the effect of Cur-B on the expression of Jagged-1 (ligand of Notch-1) and Hes-1 (downstream target). It was found that Cur-B reduced the expression of Jagged-1 by 0.83 ± 0.03, 0.76 ± 0.07, 0.50 ± 0.04, and 0.26 ± 0.05 folds, whereas this reduction for Hes-1 was found to be 0.89 ± 0.03, 0.73 ± 0.03, 0.49 ± 0.05, and 0.21 ± 0.04 folds, as compared to the vehicle or control in PCa cells (Figure 8).

## 4 Discussion

Due to delayed diagnosis and inadequacy in the therapeutic strategies, PCa still remains the prime cause of cancer-associated demises in men (Dong, et al., 2019). It has been established that multiple dietary phytochemicals have been explored for their chemo-preventive potential and their capability to suppress the growth and proliferation rate, metastasis, invasion, and angiogenesis without incurring deleterious aftereffects. Thus, they could be a plausible adjunct in declining both the occurrence and mortality of cancer by obstructing, reversing, and delaying the process of tumorigenesis (Ranjan, et al., 2019). For decades, cucurbitacins which are also called tetracyclic triterpenes frequently found in the Cucurbitaceae family are used as complementary medicine (Budisan, et al., 2017). They hold the potential to be employed as plausible bioactive agents for suppressing the progression of cancer, and also, these compounds contain various structural modifications for anticipatory chemotherapeutic modalities. Henceforth, we studied and explored the mechanistic action and potential utility of Cur-B in PCa cells with the urge of finding a new and potent anticancer candidate. In the present investigation, we have demonstrated that Cur-B exhibited an anticancer effect, enhanced the generation of intracellular ROS, and apoptosis induction via downregulating Notch signaling pathways in PCa cells.

First, we demonstrated that treatment with Cur-B significantly suppressed the growth and proliferation of LNCaP PCa cells in a dose-proportional manner as assessed by the MTT assay. Thereafter, phase contrast microscopy has revealed that treatment of Cur-B has substantially altered the morphology of LNCaP cells by detaching them from the surface of flasks, rounding up of cells, and cellular constriction, which indicated Cur-B-mediated cytotoxicity in PCa cells.

Earlier investigations have suggested that various chemotherapeutic candidates suppress cellular proliferation by inducing apoptosis (Chen, et al., 2012). It is very well-known that chromatin condensation and fragmentation are the chief characteristics of apoptosis, which are associated with cell rounding, the decline in the volume of cells, and the formation of apoptotic bodies (Wong, 2011). Our results of DAPI staining suggested that Cur-B instigated apoptosis in androgen-dependent LNCaP cells. Furthermore, our results of PI staining also support the aforementioned results, where apoptotic nuclei stained with PI in LNCaP cells indicated that Cur-B induced apoptosis in PCa cells.

During apoptosis caspases (proteolytic enzymes) are activated from their inactive proenzymes form to their active form, which eventually leads to cleavage at specific aspartate sites. However, caspase-8 and -9 are initiators which undergo autocatalytic activation and mediate the procession of executioner-caspase-3 (Shi, 2004). It was observed that the treatment of Cur-B mediates the dose-dependent activation of caspase-8, -9, and -3, which indicated that Cur-B mediates apoptosis induction via both extrinsic and intrinsic apoptotic pathways in human PCa cells. Moreover, Cur-B-induced cytotoxicity in LNCaP cells was substantially decreased and reduced by caspase-8, -9, and -3 inhibitors, thereby implicating the critical contribution of caspase-8, -9, and -3 during Cur-B-induced apoptosis. Thus, it is can be reasonably concluded that Cur-B might induce apoptosis in LNCaP cells via both caspase-dependent and independent pathways.

An upsurge in oxidative stress via the elevation of intracellular ROS levels is anticipated to modulate cellular homeostasis resulting in a decrease in the dissipation of mitochondrial membrane potential and DNA damage, eventually leading to apoptosis (Guo, et al., 2013). Moreover, DCHF-DA fluorescence staining substantiated that Cur-B mediates the augmentation of intracellular ROS in LNCaP cells. In addition, the quantitative evaluation of the intracellular ROS level was in support of microscopy and thus concluded that Cur-B induces ROS-mediated apoptosis in LNCaP cells.

Mitochondria are the chief cellular sites for the production of ROS which results in the loss of mitochondrial membrane potential leading to cytochrome c release (Zorov, et al., 2014). Our findings revealed that Cur-B treatment decreases the NIR fluorescence, which was indicative of mitochondrial depolarization in Mito-NIR dye-stained LNCaP PCa cells. Thus, Cur-B treatment effectively altered the mitochondrial membrane potential with increasing doses of Cur-B in PCa cells.

Various phases of cell cycle progression in cancer cells are positively regulated by cyclin-dependent kinases (CDKs) and negatively regulated by CDK inhibitors (CDKIs) (Shi, 2004). Our qPCR analysis revealed that treatment of Cur-B modulated the gene expression of cyclin D1, CDK4, and p21 in prostate cancer cells. Furthermore, Bcl-2 family members are also known as the regulators of apoptosis. Both pro- and anti-apoptotic proteins act in coordination to maintain the homeostatic balance within the cells (Moin, et al., 2021). Cur-B treatment significantly elevated the mRNA expressions of Bax and Bad and deflated the levels of Bcl-2 expression in LNCaP PCa cells.

Hyperactivated Notch signaling is found to be associated with various carcinomas of the breast, brain, cervical, lung, colon, head, and neck, renal cell carcinoma, acute myeloid, Hodgkin and large cell lymphomas, and pancreatic cancer (Singh, et al., 2019). However, Notch-1 is reportedly found to be overexpressed in prostate cancer cells (Guo, et al., 2011). Therefore, in the present investigation, our findings revealed that Cur-B obstructed the Notch signaling pathway in PCa cells. It was observed that gene expression studies have shown that Cur-B downregulated Notch signaling via the inhibition of Notch-1 and its ligand Jagged-1 within treated PCa cells along with the suppression of Hes-1, its downstream target. It is essential to target Notch as it may have chemo-preventive and anti-cancer effects, which could lead to decreased incidences of disease and ameliorated survival rate of patients. Although there are multiple drug candidates or therapeutic agents in the process to accomplish this goal, the Notch signaling pathway is often found to be hyperactivated in solid tumors and regulates cell fate decisions and maintains cancer stem cells. Additionally, the enhanced expression of Notch pathway components is clinically linked with poorer prognosis in various types of cancer. As a consequence, targeting Notch may have chemopreventive and anti-cancer effects, leading to decreased incidences of diseases and improved survival. However, various researchers are paying more attention in developing such therapeutic agents to achieve this goal. Utilizing the potential of naturally derived agents to target the Notch pathway offers the potential advantage of low toxicity to normal tissue. There are various terpenoids, such as glycyrrhizin, carvacrol, and andrographolide, which modulate Notch pathway components in a context-dependent manner. A recent study showed that the inhibitory effect of carvacrol on the cell cycle is associated with the downregulation of cyclinD1 and CDK4 and the upregulation of suppressor protein p21 in androgen-independent PC-3 human prostate cancer. Additionally, carvacrol blocked Notch-1 and Jagged-1 protein expression, resulting in the inhibition of the Notch signaling pathway (Khan et al., 2019). In addition, andrographolide is a lactone diterpenoid that constraints the growth of SW-480 cells through the inhibition of the Notch signaling pathway (Khan et al., 2021). Furthermore, glycyrrhizin, a triterpenoid, suppressed the growth of cervical cancer cells via targeting the key components of the Notch signaling pathway (Ahmad et al., 2022). Thus, terpenoids modulate Notch signaling in several types of cancer and concurrently decrease *in vitro* cell viability and *in vivo* tumor growth, suggesting a potential role for their clinical use to target Notch pathway components, either alone or in combination with current therapeutic agents.

Consequently, various scientists have switched their focus toward dietary natural compounds for targeting the Notch signaling cascade in cancer (Kiesel and Stan, 2022). Therefore, our present study strengthened the inhibitory role of Cur-B against the development and progression of PCa via the induction of apoptosis and alteration of the Notch signaling cascade. Furthermore, there are some shortcomings in utilizing Cur-B as an effective anticancer drug in which it exerts non-selective toxicity which indicates its toxic nature toward normal cells. However, under conditions of a reasonable dose and administration time, Cur-B exhibits no toxicity to experimental cells and animals. Various pharmacokinetic studies have demonstrated that Cur-B can be absorbed and cleared *in vivo* and is broadly distributed in internal organs in huge amounts with a high tissue to plasma ratio; however, its oral bioavailability is low (Dai et al., 2023).

## 5 Conclusion

To summarize, our report revealed that Cur-B holds a propitious apoptotic and anticancer potential against the androgen-dependent LNCaP cell line *in vitro*. Our results concluded that Cur-B suppressed the growth of PCa cells, which is associated with apoptosis induction, caspase activation, ROS generation, dissipation of mitochondrial membrane potential, and modulation of genes associated with apoptosis and cell cycle arrest. Thus, our data affirmed the plausible potential of Cur-B as an alternative and complementary medicine for the treatment of PCa.

## Data Availability

The raw data supporting the conclusion of this article will be made available by the authors, without undue reservation.
