# Corrigendum: Cucurbitacin-B instigates intrinsic apoptosis and modulates Notch signaling in androgen-dependent prostate cancer LNCaP cells

**DOI:** 10.3389/fphar.2023.1286507

**Published:** 2023-09-29

**Authors:** Ahmed Alafnan, Nasrin E. Khalifa, Talib Hussain, Mhdia Elhadi Osman

**Affiliations:** ^1^ Department of Pharmacology and Toxicology, College of Pharmacy, University of Ha’il, Ha’il, Saudi Arabia; ^2^ Department of Pharmaceutics, College of Pharmacy, University of Ha’il, Ha’il, Saudi Arabia; ^3^ Department of Pharmaceutics, Faculty of Pharmacy, University of Khartoum, Khartoum, Sudan; ^4^ Department of Clinical Pharmacy, College of Pharmacy, University of Ha’il, Ha’il, Saudi Arabia

**Keywords:** anti-cancer, apoptosis, cell cycle, cucurbitacin-B, Notch signaling

In the published article, there was an error in the **Funding** statement. The correct Funding statement appears below.

“This research has been funded by Deputy for Research and Innovation, Ministry of Education through Initiative of Institutional Funding at University of Ha’il—Saudi Arabia through project number IFP-22 112”.

The authors apologize for this error and state that this does not change the scientific conclusions of the article in any way. The original article has been updated.

