# Supplementary figures and images for: Cucurbitacin-B instigates intrinsic apoptosis and modulates Notch signaling in androgen-dependent prostate cancer LNCaP cells
Source: Front Pharmacol. 2023 Jun 28;14:1206981. doi: 10.3389/fphar.2023.1206981 (PMC10338038; doi:10.3389/fphar.2023.1206981)

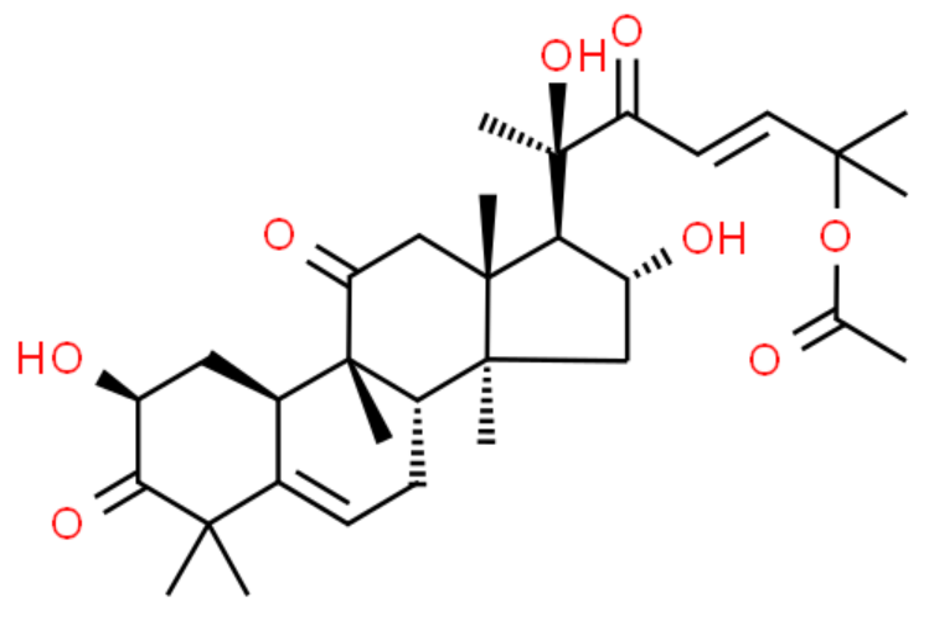

Supplement: Supplementary file 1 [file Image1.TIF]
